# Transplantation of autologous extracellular vesicles for cancer-specific targeting

**DOI:** 10.7150/thno.51344

**Published:** 2021-01-01

**Authors:** Alessandro Villa, Mariangela Garofalo, Daniela Crescenti, Nicoletta Rizzi, Electra Brunialti, Andrea Vingiani, Paolo Belotti, Carlo Sposito, Silvia Franzè, Francesco Cilurzo, Giancarlo Pruneri, Camilla Recordati, Chiara Giudice, Alessia Giordano, Monica Tortoreto, Giangiacomo Beretta, Damiano Stefanello, Giacomo Manenti, Nadia Zaffaroni, Vincenzo Mazzaferro, Paolo Ciana

**Affiliations:** 1 Department of Health Sciences, University of Milan, Milan, Italy.; 2 Department of Oncology and Hemato-Oncology, University of Milan and Pathology and Laboratory Medicine, Istituto Nazionale Tumori IRCCS Foundation (INT), Milan, Italy.; 3 Department of Oncology and Hemato-Oncology, University of Milan and HPB Surgery and Liver Transplantation, Istituto Nazionale Tumori IRCCS Foundation (INT), Milan, Italy.; 4 Department of Pharmaceutical Sciences, University of Milan, Italy.; 5 Department of Veterinary Medicine, University of Milan, Milano, Italy.; 6 Molecular Pharmacology Unit, Department of Applied Research and Technological Development, Fondazione IRCCS Istituto Nazionale Tumori, Milan, Italy.; 7 Department of Environmental Science and Policy, University of Milan, Milan, Italy.; 8 Animal Health and Welfare Unit, Department of Applied Research and Technical Development, Fondazione IRCCS Istituto Nazionale Tumori, Milan, Italy.

**Keywords:** biocompatible nanoparticles, cancer imaging, theranostic agents, drug delivery, fluorescence

## Abstract

Nano- and microsized extracellular vesicles (EVs) are naturally occurring cargo-bearing packages of regulatory macromolecules, and recent studies are increasingly showing that EVs are responsible for physiological intercellular communication. Nanoparticles encapsulating anti-tumor theranostics represent an attractive “exosome-interfering” strategy for cancer therapy.

**Methods**: Herein, by labeling plasma-derived EVs with indocyanine green (ICG) and following their biodistribution by *in vivo* and *ex vivo* imaging, we demonstrate the existence of nanoparticles with a highly selective cancer tropism in the blood of colorectal cancer (CRC) patients but not in that of healthy volunteers.

**Results**: In CRC patient-derived xenograft (PDX) mouse models, we show that transplanted EVs recognize tumors from the cognate nanoparticle-generating individual, suggesting the theranostic potential of autologous EVs encapsulating tumor-interfering molecules. In large canine breeds bearing spontaneous malignant skin and breast tumors, the same autologous EV transplantation protocol shows comparable safety and efficacy profiles.

**Conclusions**: Our data show the existence of an untapped resource of intercellular communication present in the blood of cancer patients, which represents an efficient and highly biocompatible way to deliver molecules directly to the tumor with great precision. The novel EV-interfering approach proposed by our study may become a new research direction in the complex interplay of modern personalized cancer therapy.

## Introduction

The selective intratumor cell delivery of diagnostics and therapeutics has always been one of the main challenges of cancer therapy and still represents a major unmet need. Although many approaches have been proposed, only lipid-based formulations have had any impact in clinical practice 1, 2; however, the use of these formulations is restricted due to limitations in their drug loading capacity and tumor-selective homing 3 properties, immunogenicity and ability to penetrate tissues outside the reticulo-endothelial system, which represents an additional problem in this context 3.

Among the more “biocompatible” and efficient tools used to overcome these limitations 4, 5, extracellular vesicles (EVs) are naturally occurring cargo carriers that have recently attracted the attention of the drug delivery field 6, 7; these nanoparticles are nano- to microsized lipid membrane-bound vesicles secreted into the extracellular environment to transport proteins, lipids and nucleic acids from cell to cell 8. In recent decades, EVs have been found to be essential for intercellular communication and regulation of different biological functions under both physiological 9 and pathological conditions 10, 11. The addition of small molecules and biologics to the cargo of naturally occurring EVs was shown to enhance the anti-cancer activity of EVs in animal models 12, 13 while displaying a safe toxicological profile in patients treated with dendritic cell-derived EVs 14, 15.

Recently, we performed a series of experiments aimed at evaluating the biodistribution and therapeutic applications of tumor-derived EVs using both immunodeficient 12 and immune competent 12, 16 cancer models. These studies demonstrated that tumor-derived EVs loaded with therapeutic and diagnostic agents are biocompatible, since they did not induce systemic inflammatory reactions in immune competent models and the immune system did not affect their biodistribution 10, 16, 17. Moreover, these EVs are very versatile since they can deliver theranostic cargo selectively to neoplastic tissues, including not only tumors of the same tissue origin but also different tumor types, even from different species 12, 16, 18. Interestingly, these EVs can promote the synergistic anti-cancer effect of the delivered therapeutic agents 12. However, the clinical translation of these EVs is still hindered by the lack of information on the long term effects of exogenous tumor-derived EVs in humans and their potential for triggering undesired tumor-promoting effects 19.

To overcome these problems, we proposed to isolate EVs from cancer patients and use them in an autologous transplantation protocol aimed at delivering theranostic cargo to neoplastic tissues. Herein, we showed that EVs isolated from the plasma of colorectal cancer (CRC) patients, but not those isolated from healthy individuals, are able to recognize the cognate tumor and deliver a diagnostic fluorescent agent (indocyanine green, ICG) directly into the neoplastic tissue when administered by i.v. injection into patient-derived xenograft (PDX) mouse models. Moreover, we demonstrate that this autologous transplantation protocol can be scaled up to large canine breeds bearing spontaneous skin and breast tumors, opening the way to potential clinical applications.

## Methods

### Experimental Design

In this study, we evaluated the presence of EVs in the blood of colorectal cancer patients that displayed selective capabilities to home and deliver a fluorescent diagnostic agent (ICG) to neoplastic tissues *in vivo* in mouse models of cancer. First, after the approval of the Institutional Ethic and Scientific Review Board and receiving signed informed consent of all the patients, patient-derived extracellular vesicles (PDEVs) were isolated from the plasma of CRC patients (stage IV) using differential ultracentrifugation. Differential ultracentrifugation involves serial stepwise centrifugation to remove components other than EVs and is one of the most widely used EV-separation and concentration techniques, according to the International Society of Extracellular Vesicles (MISEV 2018). A disadvantage of this isolation technique is that it may collect more impurities compared to other techniques based on filtration. However, in previous experiments we tried two strategies: increasing the number of washing/ultracentrifugation steps and filtering the EVs. The first strategy was successful since the tumor tropism of the EVs was preserved, and this strategy was used to extensively purify the samples (e.g., for proteomic studies); however, ultrafiltration did not give good results since the EVs lost their tumor homing capacity using this method. Therefore, for the purpose of the autologous tumor recognition protocol, we selected ultracentrifugation because it appears to be fast, simple, and have fewer problems for clinical translation. To ensure statistical power, 8 consecutive CRC patients of both sexes admitted at the Gastrointestinal and HPB Surgery Unit of the Istituto Nazionale Tumori IRCCS Foundation, Milan, Italy, were enrolled in this study (Table S1). The aim of recruiting a consecutive, unselected, genetically heterogeneous CRC population was intentionally aimed at testing PDEV tumor tropism capabilities independent of various clinical and pathological conditions presenting in colorectal cancer patients. PDEV tropism was initially tested on two different mouse models of carcinoma, a syngeneic model of CRC and a genetic model of breast cancer, in which PDEVs or EVs originating from healthy donors were loaded with ICG and injected i.v. 24 h prior to *in vivo* imaging sessions. This time point was selected based on our previous reports showing that at early time-points, a systemic distribution of fluorescence occurs, while at 24 h, a peak of fluorescence emission is detectable only in neoplastic tissues 16, 18. In all the *in vivo* experiments of the current work, 6- to 8-week-old mice were used. Based on the effect size determined in our previous experiments 12, 16, 18, for these preliminary experiments, we used *N* = 3-5 mice per experimental group, which enabled us to statistically assess the homing capabilities of PDEVs *versus* EVs derived from healthy donors. Then, PDEVs were tested on PDX models generated by implanting tumor fragments derived from the enrolled patients in 6- to 8-week-old female severe combined immunodeficient (SCID) mice that were homozygous for the severe combined immune deficiency spontaneous mutation. For this purpose, prior to tumor implantation, mice of similar age, size and weight were randomly assigned to the protocol. Two of the 8 groups of animals (N = 2 mice per group) were excluded due to unsatisfactory growth of the engrafted tumor cells. PDEV injections were therefore performed on a total of 6 groups, which was considered a priori to be sufficient to ensure statistical power in the case of positive detection in at least 80% of cases. Each PDX mouse was injected with PDEVs derived from the same patient from whom PDX tumor originated for the *in vivo* and *ex vivo* imaging sessions. Sampling and histology on the resected tumor specimens were conducted by human pathologists (in the Pathology Department, Istituto Nazionale Tumori IRCCS Foundation, Milan, Italy) and veterinary pathologists (at Veterinary Pathology, University of Milan, Italy) who were experts in oncology. These pathologists were not blinded when assessing PDEV homing with *in vivo* imaging but were blinded when assessing PDEV homing in histopathological samples. For the scale-up, proof-of-principle experiments on large canine breeds, 2 client‐owned dogs with a histology-confirmed diagnosis of palpable, superficial tumors (i.e.: one grade 1 mast cell tumor of the mammary region and one breast carcinoma) were enrolled and underwent surgery at the University Veterinary Hospital, Section of Veterinary Surgery, Lodi-Milan. After obtaining informed consent of the animals' owners, PDEVs were isolated from the plasma of the dogs, loaded with ICG, and injected into the animal of origin 24 h before surgical removal of the tumor. Palpability of the tumor and superficial location in the breast region, together with indication for surgical removal, were chosen to comply with ethical requirements of the intervention and to assess tumor homing capabilities of PDEVs independently from histology and biologic potential of the studied tumors.

### EV preparations from the blood of CRC patients

Venous blood (30 mL) was collected in the surgical ward, from the same i.v. line used for anesthesia induction, in 8 patients scheduled for surgical removal of CRC liver metastases and admitted to the Hepato-Pancreato-Biliary Unit of the National Cancer Institute of Milan (Istituto Nazionale Tumori, IRCCS: INT). All the patients signed a specific informed consent for blood and tumor tissue processing, aimed at EV extraction and xenograft creation, respectively, according to a specific protocol approved by the Institutional Ethics and Scientific Review Board. Blood samples were also collected from 3 healthy volunteers using the same method. The blood was immediately centrifuged at 2500 g for 15 min at room temperature to remove blood cells and prevent platelet activation and release of platelet-derived EVs. The supernatants were transferred into new tubes (the bottom 10% of the supernatant above the blood cells was discarded), and the samples were centrifuged again at 2500 g for 15 min at room temperature. The supernatants were processed by ultracentrifugation for 2 h at 100000 g at 4 °C using an Optima L-80 XP ultracentrifuge (Beckman Coulter) with rotor SW32Ti (Beckman Coulter). Supernatants were aspirated, and the EV-containing pellets were resuspended in 100 µL phosphate-buffered saline (PBS, Lonza) and stored at -80 °C before use. The EVs were loaded with ICG by incubating 1-5E08 EVs in PBS for 12 h at 4°C with 50 µg/mL ICG (Sigma). Next, the samples were centrifuged at 150000 g for 3 h to pellet the EVs. The supernatants containing unbound ICG were removed, and the pellet was washed by suspending it in PBS and pelleting it again at 150000 g. Following isolation, EV profiling was carried out in accordance with the international guidelines for EV characterization 20, 21. Based on the physical/biochemical characteristics of the different EV preparations, to standardize the isolation procedure, a number of QC ranges were experimentally defined and are listed in Table 1.

### Cell culture

The MC-38 mouse colon cancer cell line was purchased from Kerafast, USA (ENH204-FP, RRID: CVCL_B288). The cells were cultured at 37 °C and 5% CO_2_ in Dulbecco's modified eagle medium (DMEM, Lonza, Switzerland) supplemented with 10% fetal bovine serum (FBS, Gibco Laboratories, USA), 100 µl/mL 1% penicillin/streptomycin (Gibco Laboratories) and 1% L-glutamine (Gibco Laboratories). The cells were tested for mycoplasma by the provider on 6/25/2018.

### Size distribution determination by nanoparticle tracking analysis (NTA)

Size distribution and concentration of patient-derived EV formulations were analyzed by NTA using a Nanosight model LM14 (Nanosight) equipped with a blue laser (404 nm, 70 mV) and sCMOS camera. NTA was performed for each sample by recording three 90 s videos that were subsequently analyzed using NTA software 3.0 (Nanosight). The detection threshold was set to level 5 and the camera level to 15.

### Cryo-electron microscopy (EM)

Cryo-EM images were acquired with an FEI Talos Arctica 200 kV FEG electron microscope equipped with an FEI Falcon 3EC direct electron detector and Volta Phase-plate. Prior to Cryo-EV imaging, the samples were vitrified on an FEI Vitrobot IV apparatus and processed as previously reported.

### Immunoblotting

For immunoblotting, extracellular vesicles were isolated from patients' and healthy volunteers' blood according to the protocol described above. After the ultracentrifugation step, the supernatants were removed, and the EV-containing pellets were resuspended in a proper volume of 1X RIPA buffer (150 mM NaCl; 1% NP-40; 0.5% sodium deoxycholate; 0.1% SDS; 50 mM Tris-HCl, pH 8.0) supplemented with protease inhibitor cocktail (Roche).

EV protein concentrations were quantified using a Bradford assay kit (Thermo Scientific). Twenty micrograms of EV protein lysates were boiled at 95 °C for 5 min, separated on 4-10% SDS-PAGE using beta-mercaptoethanol as a reducing agent and transferred onto nitrocellulose membranes (Amersham). The membranes were then blocked in 5% nonfat dry milk in TBS-T (0.2% Tween-20) at RT and incubated overnight with primary antibodies against the exosomal markers CD63 (SAB4301607 Sigma, 1:1000), TSG101 (4A10 Abcam, 1:500) and CD9 (C9993 Sigma, 1:500). The immunoreactive bands were visualized with chemiluminescence using ECL Western Blotting Analysis System according to the manufacturer's instructions (Amersham).

### Liquid chromatography (LC)

Ten micrograms of protein lysate were diluted in 50 mM ammonium bicarbonate, reduced with dithiothreitol (DTT) at a final concentration of 5 mM for 30 min at 52 °C, and then alkylated with 15 mM iodoacetamide for 20 min in the dark at room temperature. Trypsin digestion (1/20 w/w, Roche, USA) was carried out at 37 °C overnight. The resulting peptides were used for analysis by nLC using a Dionex Ultimate 3000 nano-LC system (Sunnyvale CA, USA).

Mass spectrometry (MS) spectra were acquired in a m/z range of 375-1500 Da at 120,000 resolution. Each sample was analyzed in three technical triplicates.

### Animals

All the animal experiments were performed and approved by the Italian Ministry of Research and University (permission numbers: 12-12-30012012, 547/2015) and regulated by a departmental panel of experts. C57BL/6NCrl (Charles River, MGI: 2683688) and MMTV-NeuT (The Jackson Laboratory, MGI: 1930204) mice were maintained at the animal facility of the University of Milan under standard conditions according to institutional guidelines. SCID mice were maintained at the animal house of the Fondazione IRCCS Istituto Nazionale dei Tumori under standard conditions according to institutional guidelines. After an acclimatization period of 14 days, murine syngeneic grafts were established by s.c. injections of 1E06 MC-38 cells into the neck of 12-week-old male C57BL/6 mice. The health status of the mice was monitored daily, and as soon as signs of pain or distress were evident, the mice were euthanized. EV injections were performed when the tumor size reached a diameter of ~10 mm. For the homing tests, the mice were i.v. injected with 2-4 E09 EVs/kg.

### Patient-derived xenograft (PDX) generation

Each of the stage IV CRC patients who were enrolled underwent surgical removal of liver metastases according to clinical multidisciplinary decisions. Participation in the study did not influence the clinical treatment scheme, and the study protocol did not have any influence on the planned course of surgery or subsequent follow-up. The biological human material collected for this study was used in accordance with the Declaration of Helsinki. The experimental animals were followed in the INT-Milan certified Animal House according to international standards of animal welfare [World Organization for Animal Health (OIE)] under veterinary supervision of the Institutional and National Animal Well-being Committee, which previously approved the study protocol (Authorization 401/2019-PR).

Briefly, the following steps were followed to generate stable PDX models from CRC patients undergoing surgical removal of their tumor.

Immediately after tumor removal, in the operating room, the surgeon examined the surgical specimen and the tumor tissue contained within. After a rough assessment of cellular vitality - i.e., discarding necrotic, calcific and fibrous lesions - to minimize ischemic damage, a sample of approximately 1 cm^3^ was harvested and immediately placed in a disposable sterile vial containing MACS® Tissue Storage Solution. Patient-derived cancer xenografts were prepared on the same day of tumor harvesting. After storage of part of the tissue in liquid nitrogen for future use, the largest part of the sample was prepared in fragments of approximately 3 mm^3^ and immersed in Matrigel® for approximately 20 min. Then, 2 xenografts from each sampled tumor lesion were implanted into 6- to 8-week-old female SCID mice that were homozygous for the severe combined immune deficiency spontaneous mutation. Approximately 100 mm^3^ of fragmented tumor tissue was subcutaneously injected into the hips of the mice. After inoculation, the mice were followed daily to assess their health status and once a week to assess the growth of the inoculated mass. To assess tumor rooting and growth, implants were measured with Vernier calipers; the volume of the PDX was calculated using the formula *V*=(*d^2^* × *D*)/2, where d is the smaller diameter of the subcutaneous lesion, and D the largest diameter. To be considered properly grown and ready for the imaging experiment, the inoculated lesion had to reach the volumetric target of 300-400 mm^3^, which was approximately four-times its initial size (100 mm^3^). This criterion was met at a median of 6 weeks (range: 4-8) after the injection.

### *In vivo* and *ex vivo* imaging

*In vivo* and *ex vivo* fluorescence imaging sessions were carried out 24 h after EV treatment using the IVIS Spectrum Imaging System (PerkinElmer, Waltham, MA, USA) with suitable filters (ICG) and following the manufacturer instructions for fluorescence background subtraction. The mice were anaesthetized using isoflurane (Isoflurane-Vet; Merial, Lyon, France) and kept under anesthesia during imaging sessions carried out with the Imaging System (5 min for dorsal view and 5 min for lateral view). Fluorescence emission from selected body areas was assessed using the Living Image Software 4.6 (PerkinElmer). For *ex vivo* imaging, the mice were sacrificed by cervical dislocation. Immediately after death, selected organ imaging was also carried out. Fluorescence emission of the organs was assessed using the Living Image Software 4.6 (PerkinElmer).

### Histology and microscopy imaging

Following the *ex vivo* study, explanted tissues were fixed for histology. Briefly, selected organs were immersed in a 4% formaldehyde solution for 24 h to achieve chemical fixation. The samples were then dehydrated with ethanol, washed with xylene and paraffin-embedded. The samples were cut into 4 µm thick sections (for H&E staining), while one section of every two was cut into 10 µm thick sections for fluorescence imaging. The slides were then analyzed using a confocal microscope (Nikon A1R laser scanning confocal microscope) to acquire the fluorescence signals released by ICG and compare them to those from the adjacent sections stained with hematoxylin/eosin, acquired with a Virtual Slide Microscope (Olympus VS120). Throughout the process of fixation and paraffin-embedding, the samples were kept in the dark to avoid loss of the fluorescence signal. To assess the distribution of ICG fluorescence in the tissue sections, optical sectioning was performed by acquiring a series of focal planes 1 µm distant along the optical (z) axis of the microscope, using the z-scan mode available on Nikon A1R laser scanning confocal microscope. Orthogonal views were then reconstructed using ImageJ software (NIH).

### Autologous injection of EVs in mast cell and mammary tumor-bearing dogs

Two client‐owned male dogs with histologically confirmed diagnosis of clinically measurable grade 1 cutaneous mast cell tumor and mammary carcinoma suitable for surgical removal were enrolled at the University Veterinary Hospital - Section of Veterinary Surgery, Lodi-MI. All the animals had a single tumor location in one of their breasts, had not received previous chemotherapy, radiation, hormonal or other antineoplastic therapy, were not pregnant or lactating and did not carry any active infection or clinically significant abnormality. After obtaining the informed consent of the animals' owners, venous blood (15 mL) was collected the day before surgery, during the operation under general anesthesia and postoperatively before discharge. Twenty-four hours later, EVs were isolated from the blood according to the same protocol used for the human samples, labeled with ICG as described above, then reinfused (1 mL/min) into each dog 24 h prior to surgery. The dogs underwent unilateral mastectomy. After removal, the tumor samples were immediately transferred to the imaging facility for *ex vivo* imaging and then processed for histopathological assessment.

## Results

### Patients and isolation/characterization of plasma-derived EVs

We initially tested the hypothesis that nanoparticles released by the tumor in the bloodstream of cancer patients display the same tumor-selective tropism we previously observed in EVs produced by transformed cell lines 16. To this end, we extracted EVs from the plasma of eight CRC patients with liver metastasis (stage IV), a tumor type with features (tissue origin, invasiveness and metastasis ability) similar to those the murine MC-38 cell line from which the EVs used in previous experiments were generated 12, 16, 18. In addition, we extracted EVs from the plasma of five healthy volunteers as negative controls. All the patients had i) histological diagnosis of metastatic colorectal adenocarcinoma; ii) one or more resectable target lesions in the liver with a diameter >10 mm; and iii) presence, in the resected tumor lesion, of a vital component allowing a sample volume of approximately 1 cm^3^ for the PDX protocol. The clinical features of the patients are summarized in Supplementary Table 1, which shows a heterogeneous, unselected population of CRC-bearing patients.

**Figure 1:**
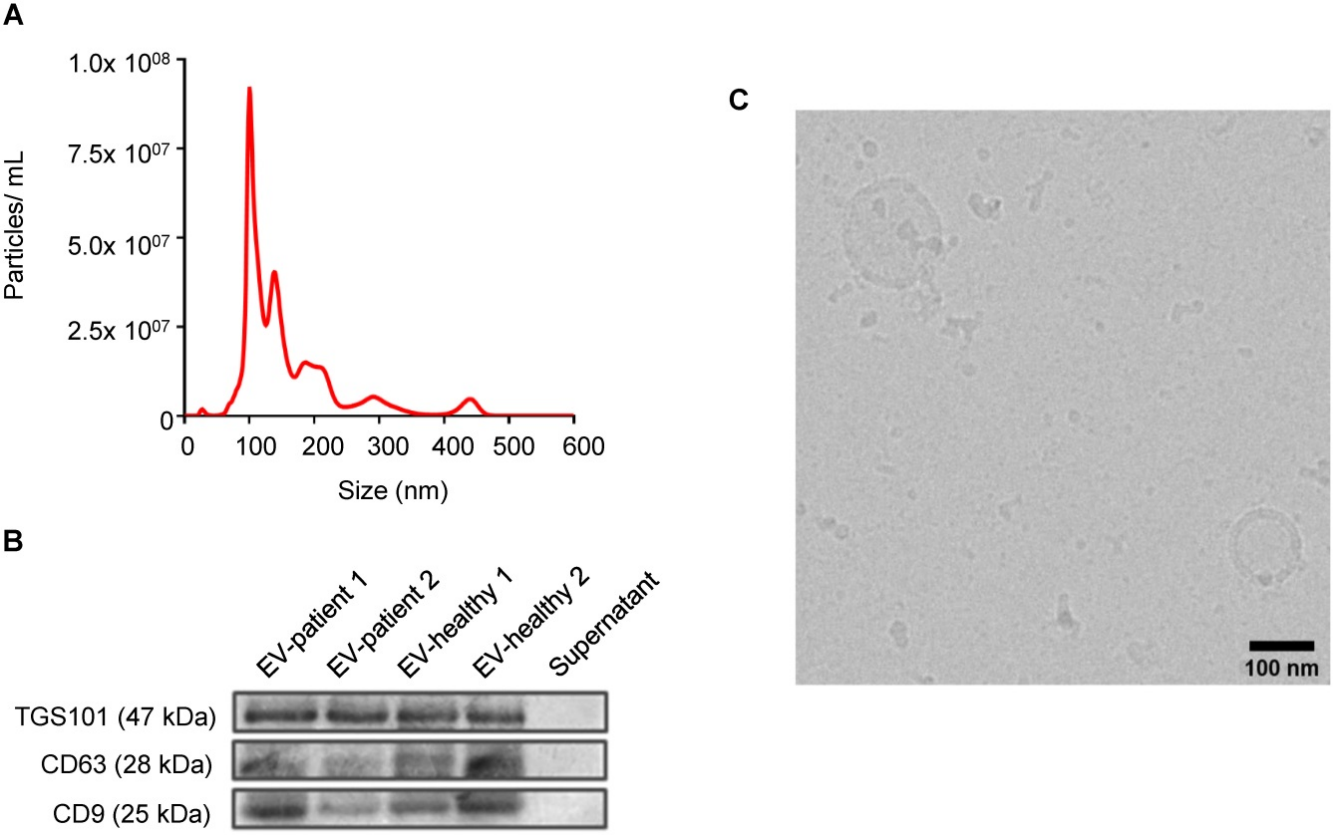
Characterization of EVs from stage IV CRC patients. All CRC patient-derived EVs displayed similar shapes, size distributions, and EV-specific marker expression. (A) Representative particle size (relating to Patient 1) distribution analysis of plasma-derived EVs obtained by nanoparticle tracking analysis (*NTA*). (B) Expression analysis of EV marker proteins Tumor Susceptibility Gene-101 (TSG101) and tetraspanin proteins (CD63 and CD9) in plasma-derived EVs from patients or healthy donors. (C) Representative EV imaging (relating to Patient 1) by cryo-electron microscopy, scale bar: 100 nm. Additional images are shown in Supplementary Figure 1.

EVs were prepared as described (see Methods). In all cases, NTA demonstrated nanoparticles of similar dimensions in the range of 50-400 nm (Figure 1A). Further characterization was carried out in accordance with the international guidelines for EV characterization 20, 21: western blot showed the presence of specific EV biomarkers, such as TGS101, CD63 and CD9 in the formulations (Figure 1B), while cryo-EM experiments directly displayed features of membrane vesicles, demonstrating EV morphology and integrity (Figure 1C, Figure S1).

### Patient-derived EVs, but not those derived from healthy volunteers, selectively target neoplastic tissue in a mouse model of colon cancer

To test the ability of plasma-derived EVs to migrate into neoplastic tissues, we evaluated their biodistribution in C57BL/6 wild type mice engrafted with the syngeneic MC-38 colon cancer cell line. Plasma-derived EVs were loaded with near infrared (NIR) fluorescent ICG; approximately 0.26 mmol ICG per mg of protein of nanoparticles were incorporated with this procedure. NTA analysis demonstrated that ICG-loaded EVs did not show any size difference between the original and the loaded particles (Figure S2). Based on the heterogeneous size reported by the NTA analysis, we defined the nanoparticles purified from patients and used in our study as EVs with a prevalence of exosomes.

**Figure 2:**
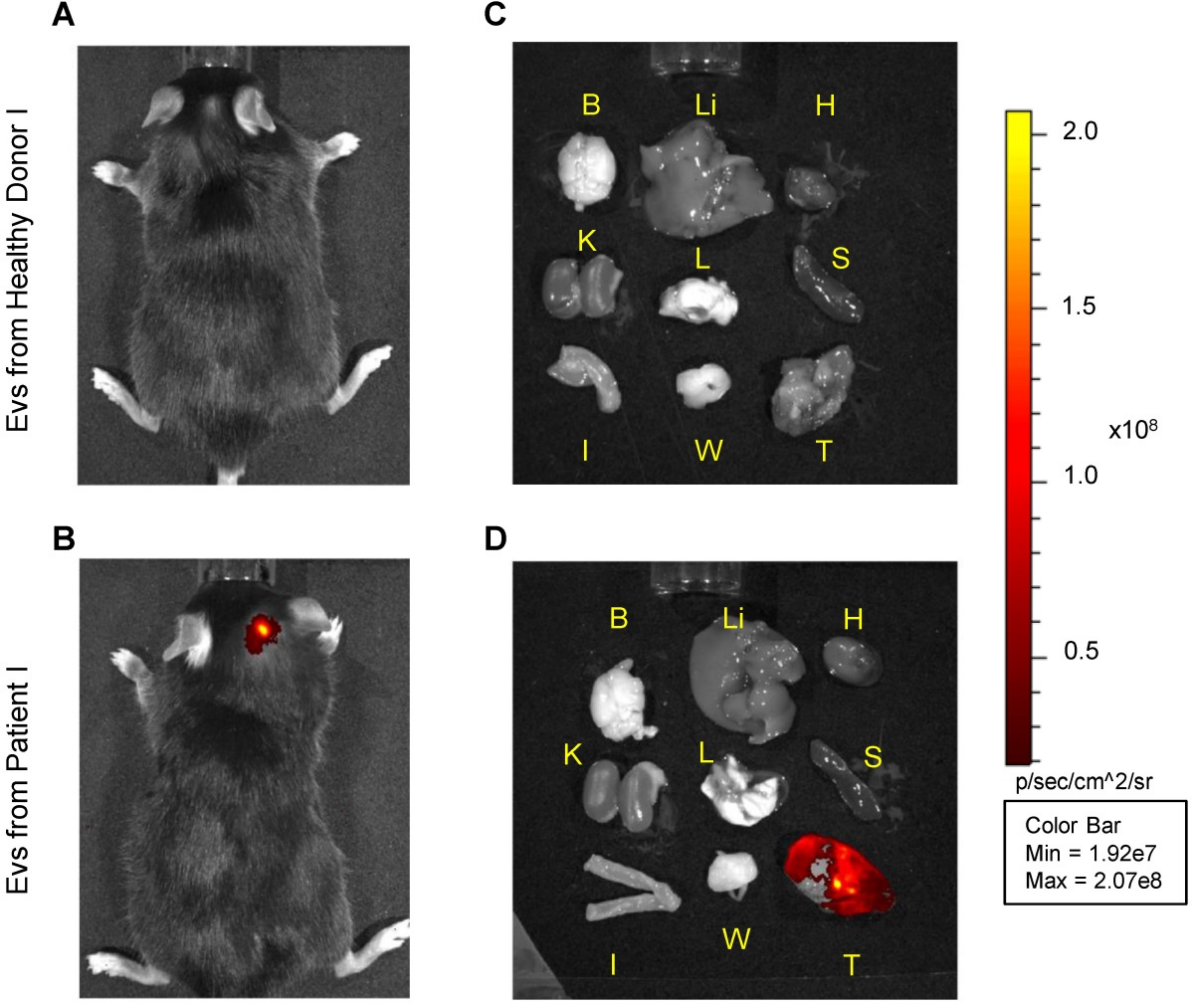
Pseudocolor representative images of ICG fluorescence. *In vivo* (left panels) and *ex vivo* (right panels) imaging of MC-38 tumor-bearing mice injected with *i*) EVs obtained from a heathy volunteer (images A and C) or *ii*) patient-derived EVs, both labeled with ICG (images B and D). Images from other experiments are shown in Supplementary Figure S3. B: Brain, Li: Liver, H: Heart, K: Kidneys, L: Lungs, S: Spleen, I: Intestine, W: White Adipose Tissue, T: Tumor.

A total of 10E08 ICG-loaded particles/tumor containing a total of 130 nmol ICG were administered by i.v. injection into a total of eight mice bearing 1 cm^3^ tumors. The biodistribution of the particles was monitored by *in vivo* and *ex vivo* imaging of the ICG fluorescence signal 24 h after treatment (Figure 2). In the mice treated with EVs isolated from the plasma of five healthy donors (Figure 2A and Figure S3), no specific fluorescence signal arising from the tumor site was detected. In most cases, a residual dim fluorescence was observed in metabolic organs, such as liver and kidneys. In contrast, mice injected with the three patient-derived EVs showed a specific signal arising from the neoplastic tissue (Figure 2B); such tumor-selectivity was confirmed by *ex vivo* imaging, showing no accumulation in the organs of mice injected with EVs obtained from heathy volunteers (Figure 2C), but specific accumulation of fluorescence was detected in the neoplasms of mice injected with patient-derived EVs (Figure 2D). Similar results were obtained in mice bearing breast tumors (Figure S3), in agreement with the previously reported heterologous tumor recognition ability of cancer-derived EVs 16.

To exclude out the possibility that some difference in the EV purification from patients and healthy donors could account for the observed differential biodistribution, additional tests were designed to further characterize possible differential serum protein (albumin and lipoproteins) contamination by LC and MS. LC analyses showed comparable protein profiles in all the isolated EVs (Figure S4A), and MS analyses revealed the presence of albumin and 10 lipoproteins in the EV preparations, and the expression of these proteins did not differ between the healthy donors and patients (Figure S4B). Finally, the ratio between the number of particles and the protein content was similar between the different preparations. This body of evidence suggests that the observed differences in the biodistribution were due to specific cancer tropism properties of the EVs isolated from CRC patients. Nonetheless, the observed tropism could have been due to specific features of the murine model of cancer not representative of human CRC, known to be genetically very heterogeneous. To exclude out this possibility as well, we investigated patient-derived EV biodistribution using patient-derived xenograft models.

### Patient derived-EVs recognize corresponding tumors grown in patient-derived xenograft (PDX) mice

**Figure 3:**
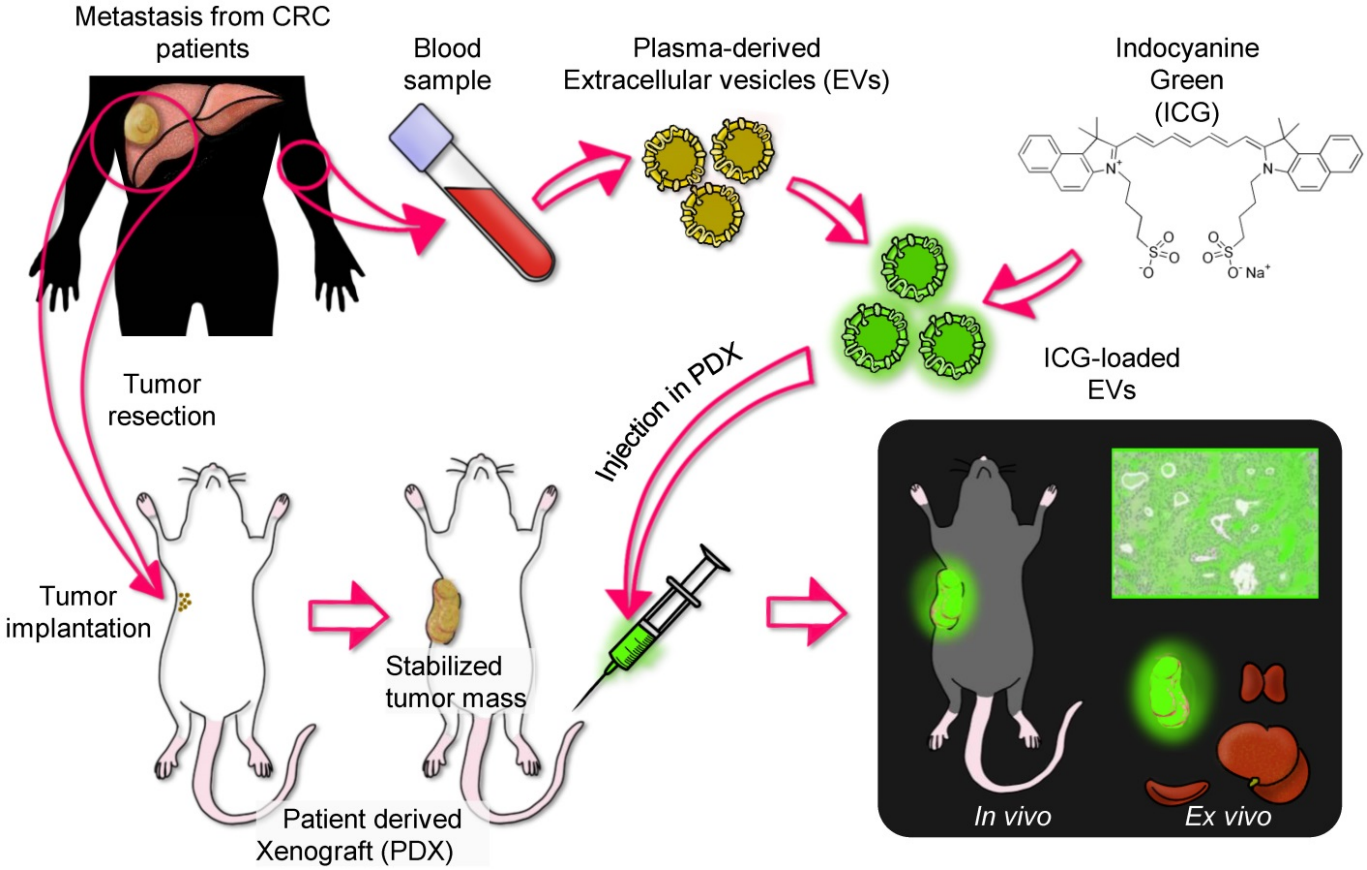
Experimental design of the testing of ICG-labeled EVs purified from CRC patients bearing liver metastasis in PDX models. Liver metastases explanted from each patient were implanted and grown in SCID mice. EVs were isolated from patient blood obtained before surgery and were loaded with ICG. When the tumor reached a volume of 300-400 mm^3^, the PDX mice were administered i.v. injections of ICG-loaded EVs (obtained from the same patient originating the tumor) and, after 24 h, were subjected to *in vivo* and *ex vivo* imaging.

**Figure 4:**
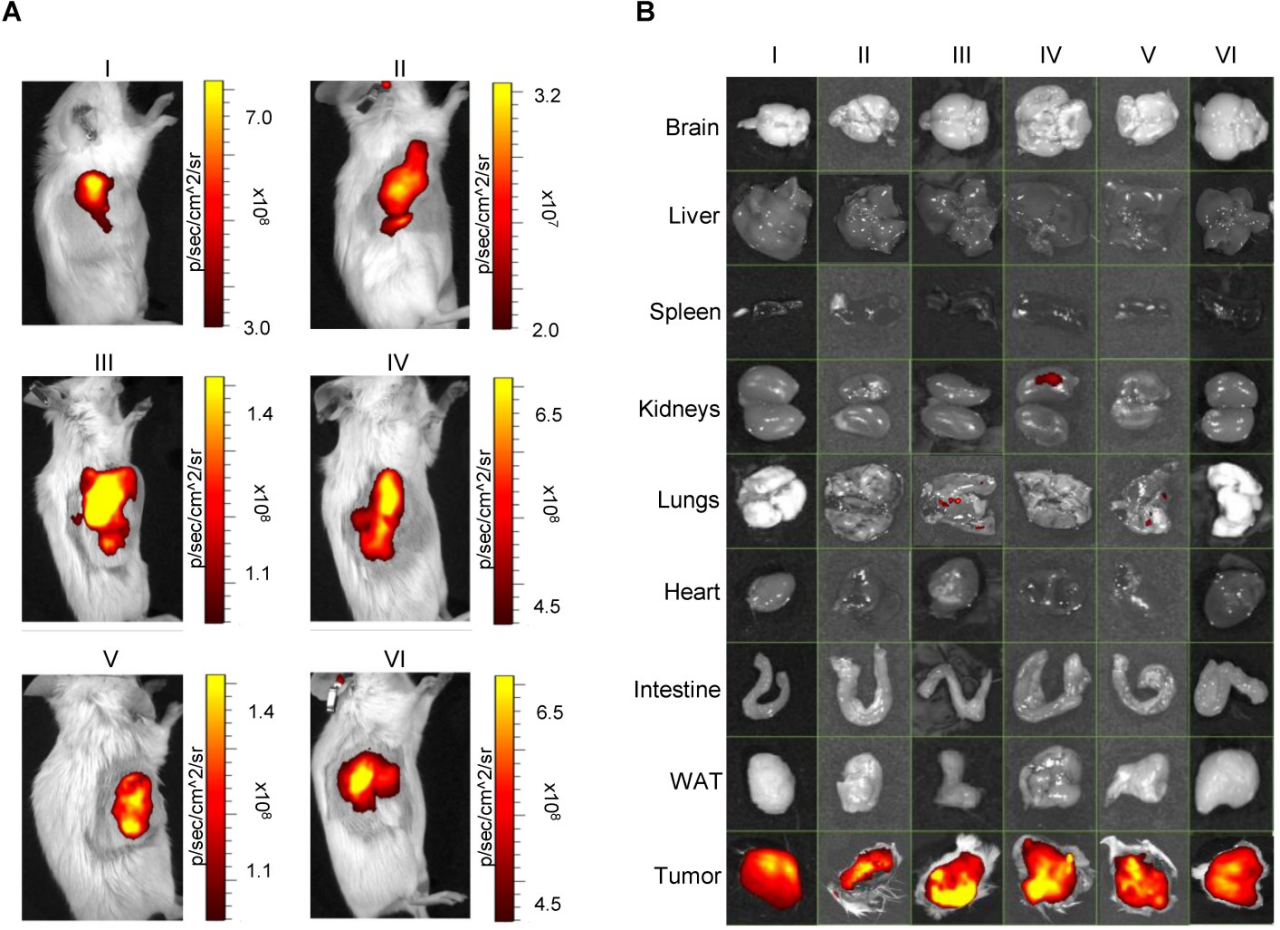
Pseudocolor representation of ICG fluorescence acquired by *in vivo* (A, left panels) and related *ex vivo* (B, right panels) imaging of isolated organs of *n*=6 PDX mice (I, II, III, IV, V, and VI) injected with EVs derived from the plasma of the same patients from which the corresponding PDX model was generated. In all cases, tumors were selectively labeled with ICG-loaded EVs. WAT: white adipose tissue.

To assess the autologous recognition ability of EVs *versus* their tumor of origin, we generated PDX models from each patient enrolled in the study in immunodeficient SCID mice and tested the homing ability of the ICG-loaded EVs isolated from the corresponding patient. Six of eight CRC samples successfully generated usable PDX tumors. The general scheme of the experiment is summarized in Figure 3. To test the homing ability, EVs loaded with ICG (EVs-ICG) were administered by i.v. injection into xenograft mice when the size of the tumor reached a volume of 300-400 mm^3^. Twenty-four hours after treatment, *in vivo* and *ex vivo* imaging was performed. *In vivo* imaging showed fluorescence accumulation in the tumor area in all six mice tested (Figure 4A). PDX-2 showed a fluorescent area in the tumor and in the retro and latero-cervical area, which was used in that animal as an alternative site of i.v. injection. The tumor tropism of ICG-loaded EVs was confirmed by *ex vivo* imaging analysis showing that fluorescence emission derived only from the neoplastic tissue (Figure 4B). Parallel experiments were performed by administering i.v. injections of free ICG (130 nmol: the same amount included as cargo into the plasma-derived EVs) into the mice, showing no specific fluorescence signal originating from the tumor (Figure S5) and confirming that the specific accumulation of fluorescence was due to encapsulation of ICG into the EVs.

**Figure 5:**
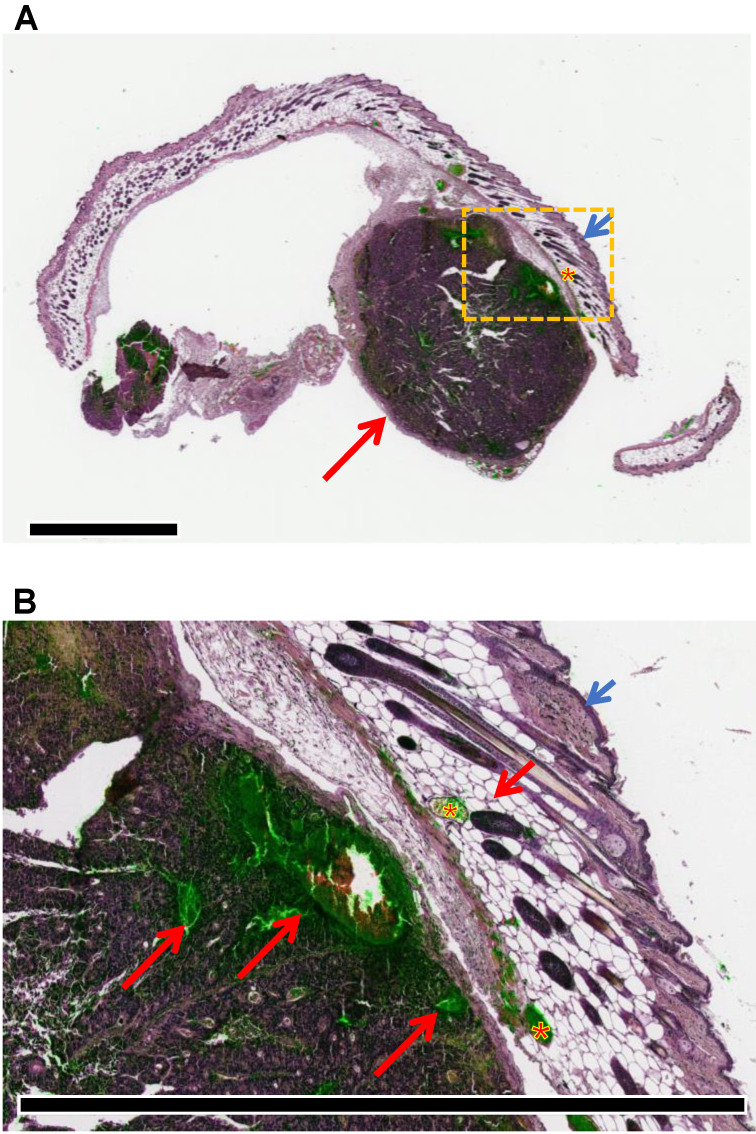
Superposition of hematoxylin-eosin staining (violet) and ICG fluorescence (green) captured from adjacent sections shows the accumulation of fluorescence signals in the tumor tissue. (A) Macroscopic view of a representative sample with the tumor nodule located underneath the subcutaneous layer (short arrow: cutis; asterisk: subscutis; long arrow: tumor). Scale bar: 2 mm. (B) Details of the tumor tissue (long red arrow: ICG accumulation in cancer cells and tumor necrosis; asterisk: ipodermal vascular spaces; short blue arrow: cutis; short red arrow: subcutis). Scale bar: 2 mm.

Microscopy analysis of hematoxylin-eosin staining and ICG-related fluorescence of adjacent sections of the removed CRC xenograft demonstrated that ICG accumulation occurred in the neoplastic tissue and not in the normal surrounding tissue (i.e., muscle-cutaneous layers) (Figure 5). While the intensity of fluorescence acquired with confocal microscopy seemed to be weaker than the signal acquired with *ex vivo* imaging, it should be noted that the latter technique acquires all fluorescence released by the tissue sample, several millimeters in size, while confocal microscopy was applied to tissue slices of a few microns thick. These data further confirmed at the microscopic level that the tumor-tropism demonstrated at the macroscopic level by *in vivo*/*ex vivo* fluorescence imaging. Moreover, orthogonal projections of focal planes acquired with confocal microscopy of the ICG sections showed distribution of the fluorescence within the tumor mass (Figure S6), consistent with previous studies demonstrating the intracellular release of the cargo by EVs 22.

The results obtained from these experiments demonstrate that patient-derived EVs can be utilized as tools for targeting patient tumors and delivering fluorescence and/or theranostics inside the tumor mass through an autologous nanoparticle infusion protocol.

### Autologous injection of EVs derived from mast cell and mammary tumor-bearing dogs recognize the corresponding tumor

Next, we asked whether the autologous transplantation protocol could be safely scaled up in a large mammalian species bearing spontaneous tumors. To this end, we isolated EVs from the blood of two dogs weighing 28 and 27 kg: one with a mast cell tumor (i.e., superficial tumors originated from the mast cells of the connective tissues) and one with a mammary tumor. After isolation and ICG-labeling of the nanoparticles as described for the human EVs, reinfusion was performed in the cognate donor dog 24 h prior to surgery. In detail, 2-4 E06 EVs/kg were suspended in 10 ml of saline and delivered at a rate of 1 mL/min.

**Figure 6:**
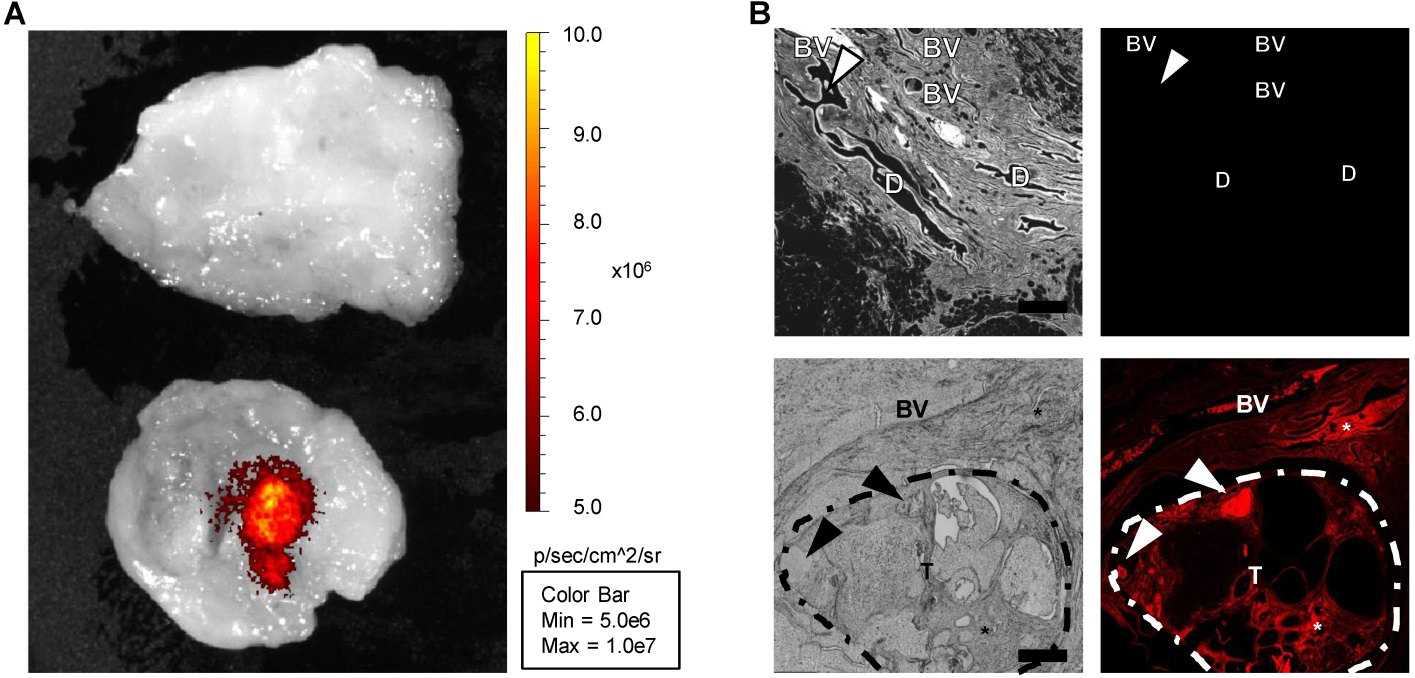
Imaging of canine control and tumor tissues. (A) *Ex vivo* imaging by the IVIS instrument revealed that, while virtually no fluorescence emission in the NIR region was detectable in the resection margin (upper sample), an area clear of any cancer cells, a fluorescence signal released by ICG could be detected in the tumor area (lower sample). (B) Bright field (gray, left panels) and ICG fluorescence (red, right panels) microscopy images, captured from slices obtained from the resection margin within a region of normal mammary tissue (upper panels) or an area within the neoplastic tissue (lower panels), confirmed the *ex vivo* imaging results. BV: blood vessel; D: mammary duct; T: tumor; asterisk: interstitial infiltration of macrophages; arrowhead: intraluminal secretory material. Scale bar: 500 µm.

Immediately after removal, the tumors were transferred to the imaging facility for *ex vivo* imaging, then fixed and paraffin-embedded for NIR imaging and histology confirmation. *Ex vivo* imaging with the IVIS instrument revealed the presence of a fluorescence signal in the NIR spectrum only in the tumor region (Figure 6A). In addition, microscopic fluorescence examination (by means of NIR filters to detect ICG fluorescence) revealed that while samples collected in the nontumor, cancer-free tissue did not exhibit detectable specific fluorescence emission, the tumor region exhibited fluorescence in the ICG spectrum (Figure 6B, Figure S7), demonstrating that ICG-loaded EVs are able to specifically target the autologous neoplastic tissue in large canine breeds with spontaneous tumors. Similar results were obtained with the dog bearing a mast cell tumor (Figure S8).

Hematologic, hepatic and renal functions were assessed before and after surgery (Supplementary Table S2), showing no significant changes. No adverse events were registered in the treated animals according to the common terminology criteria for adverse events [VCOG-CTCAE v.1.1] 23.

## Discussion

As tumor-selective drug delivery tools, EVs provide significant advantages due to their good biocompatibility, absent tumorgenicity, low immunogenicity and improved tissue-targeting potential 24. In the current work, we demonstrated the existence and isolation of tumor-specific (pathotrophic) EVs (PDEVs) in the blood of patients with stage IV (metastatic) CRC undergoing surgical tumor resection. The isolated EVs can be used for an innovative “exosome-interfering approach” involving autologous i.v. transplantation with subsequent delivery of theranostic agents 25-27 to the same tumor from which the EVs were derived. This approach stems from our previous observations showing a generalized tropism of tumor-derived EVs for neoplastic tissues, even from different species 16; in the present study, we initially made use of an immunocompetent mouse model of cancer to demonstrate the tumor targeting ability of PDEVs, a feature that was not present in EVs derived from healthy donors; the immune evasion properties of PDEVs might be ascribed to the suppression of the antigen-specific and nonspecific immune responses previously reported for tumor-derived EVs 28-30. Then, to illustrate utility for future clinical applications, we tested PDEVs in PDX mice, a preclinical model closer to humans. In the PDX model, the “exosome-interfering approach” demonstrated to be effective, selective and nontoxic and, in the case of canine large breeds with spontaneous mammary and mast cell cancers, the scaled-up dosing of ICG-loaded PDEVs was confirmed to be selective and safe. Because autologous EVs are fully biocompatible with the subject and the tumor from which they are derived, the described autologous protocol is expected to abolish the risk of immunogenicity and further increase the targeting potential of these nanoparticles compared with previously proposed EVs of heterologous origin 24. Should adequate GMP EV isolation be available, the presented strategy suggests the potential of novel approaches of personalized diagnosis and therapies in cancer patients. For instance, in surgical oncology, autologous EVs could promote the accumulation of ICG, a diagnostic reagent approved for use in humans, in tumor cells for several clinical imaging applications due to its safety profile. Indeed, ICG-loaded EVs could work as *in vivo* tumor-selective markers, guiding more precise treatment of cancer lesions. Augmented-reality devices that take advantage of such effects could improve surgical precision, achieving tumor-free margins and ultimately obtaining improved local control of cancer 31. The current practice of intra/peri-tumor direct injection of ICG to stain local lymph nodal drainage in some tumors 27 could be largely surpassed by the demonstrated specificity of ICG-loaded EVs. In addition, since EVs can be loaded with both small and macro molecules 25, 26, ICG could be codelivered with antineoplastic agents able to target even single cells and/or minimal residual disease left after surgical/loco-regional interventions or overlooked by diagnostic techniques. The use of ICG presents limitations, which should be taken into account for its use in fluorescence-guided surgery. First, as with all fluorescence imaging techniques, the fluorescence of ICG is influenced by the distance between the tumor and the imaging system, and it is therefore less effective for deeper tissue tumors 32; nevertheless, combined modalities using intraoperative ICG fluorography and ultrasonography enhance the detection capability. Furthermore, rapid elimination of ICG from the body requires the administration of high doses; however, high concentrations of ICG may lead to attenuation of fluorescence due to a quenching phenomenon. Therefore, for intraoperative application, a fine control of the dye administration/accumulation in the target tissues is also required for optimal results for ICG-loaded EVs 33. Finally, ICG is predominantly present in the circulating blood and as such, may “bleed” during the tumor mass excision, making the margins less clear when cutting 34; when encapsulated into EVs, the dye cannot be detected in the blood since it is mostly accumulated in the tumor mass, overcoming this limitation.

Our approach is not limited to diagnostic applications, and we may speculate that this “exosome-interfering approach” 35 will be further exploited by reprogramming the content of nanoparticles to (co)deliver many kinds of tumor-interfering molecules, such as biologics, oncolytic viruses and chemotherapeutic agents 12, 27, thus generating novel therapeutic strategies that may convey a more favorable efficacy and toxicity profile to conventional anti-cancer drugs. This approach has the potential to change the scenario of medical oncology. For instance, based on their selective tumor tropism, EVs may supply a very high local concentration of cytotoxic drugs, up to levels that, to date, have been precluded by the systemic toxicity of several chemotherapeutics. In line with this hypothesis, previous preclinical experiments in murine xenograft models of cancer suggest that encapsulating chemotherapeutic agents in EVs increased their efficacy 12, 36 without compromising safety 18 due to their limited delivery in tissues other than the tumor. Our own experience demonstrated that loading EVs with anti-cancer treatments (e.g., paclitaxel and oncolytic viruses) did not show alterations in the tumor-tropism features of cancer-derived EVs 12, 18; therefore, no impediment to this application for PDEVs should be expected. The clinical use of autologous EVs may be initially explored in CRC patients but also in lung, breast 16, 18 and likely in many other solid cancers, irrespective of their site of origin.

All these potentially relevant applications of autologous EVs are currently limited by the lack of knowledge of their homing mechanism and of crucial information including i) the cellular origin of patient-derived EVs, ii) the magnitude of plasma-derived EVs with tumor tropism and iii) the molecular determinants responsible for tumor tropism.

The origin of these PDEVs has not yet been determined, but based on our previous experiments, we hypothesize that the nanoparticles are released by the tumor cells themselves. Indeed, a series of experiments aimed at evaluating biodistribution and therapeutic applications of EVs produced by tumor cell lines, including the MC-38 (of murine colorectal cancer origin, 16, 18), LL/2 (of murine lung cancer origin, 16), and A549 (of human lung cancer origin, 16) cell lines, showed a biodistribution pattern that is superimposable to that observed using patient-derived EVs. Certainly, this is only circumstantial evidence, and specific experiments would be necessary to finally address this issue. However, at present, we cannot exclude the possibility that cells from the tumor microenvironment or from the blood (e.g., platelets) could release these EVs.

The main obstacle to be addressed in translating this technique to human is that, to date, the use of autologous EVs may pose a limitation in terms of the number of vesicles that can be obtained from a single blood draw. In preclinical experiments, we isolated EVs starting from 30 mL of whole blood (15 mL for canine experiments), which allowed us to obtain a volume of plasma accounting for approximately 50% of the starting volume (~15 mL from humans and ~8 mL for dogs). This plasma volume yielded 1-5E10 EVs, which - when labeled with ICG - precisely tagged tumors grafted in mice weighting 20-30 grams and allowed *in vivo* imaging of tumor tissue. Similar amounts of EVs were proven sufficient for labeling spontaneous tumors in dogs weighting approximately 30 kg: the quantity of ICG delivered to the tumor was sufficient to observe the fluorescence signal using a cooled charged-coupled device camera or a confocal microscope but was still not enough for effective intraoperative imaging. To overcome this limitation, we are considering isolating autologous EVs from plasma obtained using therapeutic exchange procedures (plasmapheresis). With plasmapheresis, it will be possible to obtain 50- to 100-fold more EVs compared to the current protocol we used for preclinical research. We hope this will ensure a massive accumulation of ICG at the tumor site, allowing intraoperative imaging of tumor margins.

Concerning the mechanism of tumor tropism, we are presently comparing the protein profiles of EVs derived from the plasma of cancer patients with those of EVs obtained from healthy donors in search for specific features of cancer-derived EVs possibly involved in the tropism of this specific population of nanoparticles. While no differences existed in expression of the reported lipoproteins (Figure S4B), we were able to identify several proteins that were differentially expressed between the two groups. Furthermore, we cannot presently exclude the possibility that RNAs 37 or metabolite cargo could play a role in the homing process. The data are still preliminary and will certainly be the subject of future investigations. Identification of molecular features of EVs is expected to optimize the use of autologous EVs through the isolation of those “active” nanoparticles with tumor tropism from the rest of circulating EVs 38, paving the way for the future generation of synthetic vesicles loaded with theranostics as a unique cargo. The present work provides compelling evidence that the biocompatible and efficient tumor targeting properties of autologous EVs, together with the simple preparation that avoids several steps of purification and potentially harmful modifications of the biologic material to be transplanted back to the patient, have the potential to greatly improve previous attempts to use these nanoparticles in cancer therapy.

The presence of consistent amounts of EVs with cancer tropism isolated from the blood of humans with advanced colorectal cancer, as well as from animals with spontaneous tumors at early stages, is consistent with previous findings demonstrating that tumor-released EVs are able to colonize distal tissues and accumulate at premetastatic niches 39. In this context, it should be noted that a strong fluorescence signal was also present in the lungs of the mouse shown in Figure S5. This observation is quite interesting because the lungs are a common site of metastasis for colorectal cancer 40. Accordingly, a faint fluorescence signal can be observed in the lungs of PDX mice III and V as reported in Figure 4. We hypothesize that the strong fluorescence present in the lungs of the mouse shown in Figure S5 could be related to a metastatic cancer stage as exhibited by the large size of the tumor, which can be seen from the *in vivo* imaging reported in the same figure (and which was comparable with the size of the tumor present in the cognate animal treated with free ICG). Unfortunately, we did not perform histological investigations to support the existence of metastatic foci in the lungs, and specimens were not collected to allow retrospective studies but will certainly be investigated in future studies.

Our findings certainly suggest a more complex role for EVs in intercellular communication at all tumor stages and across species that needs to be investigated with specific studies.

Furthermore, comparing EVs of cancer vs. noncancer origin deserves investigation, considering that EV applications are currently envisaged as the “liquid biopsy of the future” in many fields of medicine and particularly in hepatology 41, transplantation 42, inflammation, autoimmunity, organ ischemic injury, etc. 43.

The strength of the exosome-interfering strategy of EVs lies in its unique combination of robustness and diversity 43. As demonstrated in the presented experience, the biologic properties of EVs - particularly at the level of intercellular communication and through their cancer tropism after autologous transplantation - may become a new research direction in the complex interplay of modern personalized cancer therapy.

## Supplementary Material

Supplementary figures and tables.

## Figures and Tables

**Table 1:** Established QC ranges for PDEV-ICG preparations

	Blood volume (mL)	Plasma volume (mL)	No of particles/mL	Total proteins (µg)	Dv(10) (nm)	Dv(50) (nm)	Dv(90) (nm)	ICG encapsulated (µmol)
QC reference range	13.5-16.5	6-9	1E10-6E10	100-140	60-80	90-120	220-250	12-80
