# Supplementary material for: Transplantation of autologous extracellular vesicles for cancer-specific targeting
Source: Theranostics. 2021 Jan 1;11(5):2034–47. doi: 10.7150/thno.51344 (PMC7797692; doi:10.7150/thno.51344)
Supplement: Supplementary file 1 — Supplementary figures and tables. [file thnov11p2034s1.pdf]

Transplantation of autologous extracellular vesicles for cancer-specific targeting.

Authors
Alessandro Villa[1§], Mariangela Garofalo[1§#], Daniela Crescenti[1], Nicoletta Rizzi[1], Electra Brunialti[1], Andrea Vingiani[2], Paolo Belotti[3], Carlo Sposito[3], Silvia Franzè[4], Francesco Cilurzo[4], Giancarlo Pruneri[2], Camilla Recordati[5], Chiara Giudice[5], Alessia Giordano[5], Monica Tortoreto[6], Giangiacomo Beretta[7], Damiano Stefanello[5], Giacomo Manenti[8], Nadia Zaffaroni[6], Vincenzo Mazzaferro[3]*, Paolo Ciana[1]*

Affiliations
[1] Department of Health Sciences, University of Milan, Milan, Italy
[2] Department of Oncology and Hemato-Oncology, University of Milan and Pathology and Laboratory Medicine, Istituto Nazionale Tumori IRCCS Foundation (INT), Milan, Italy
[3] Department of Oncology and Hemato-Oncology, University of Milan and HPB Surgery and Liver Transplantation, Istituto Nazionale Tumori IRCCS Foundation (INT), Milan, Italy
[4] Department of Pharmaceutical Sciences, University of Milan, Italy
[5] Department of Veterinary Medicine, University of Milan, Milano, Italy.
[6] Molecular Pharmacology Unit, Department of Applied Research and Technological Development, Fondazione IRCCS Istituto Nazionale Tumori, Milan, Italy.
[7] Department of Environmental Science and Policy, University of Milan, Milan, Italy
[8] Animal Health and Welfare Unit, Department of Applied Research and Technical Development, Fondazione IRCCS Istituto Nazionale Tumori, Milan, Italy.

[§]Equally contributed

# Current address: Department of Pharmaceutical and Pharmacological Sciences, University of Padova, Italy

[*]Co-corresponding authors

paolo.ciana@unimi.it

vincenzo.mazzaferro@unimi.it

Supplementary Materials

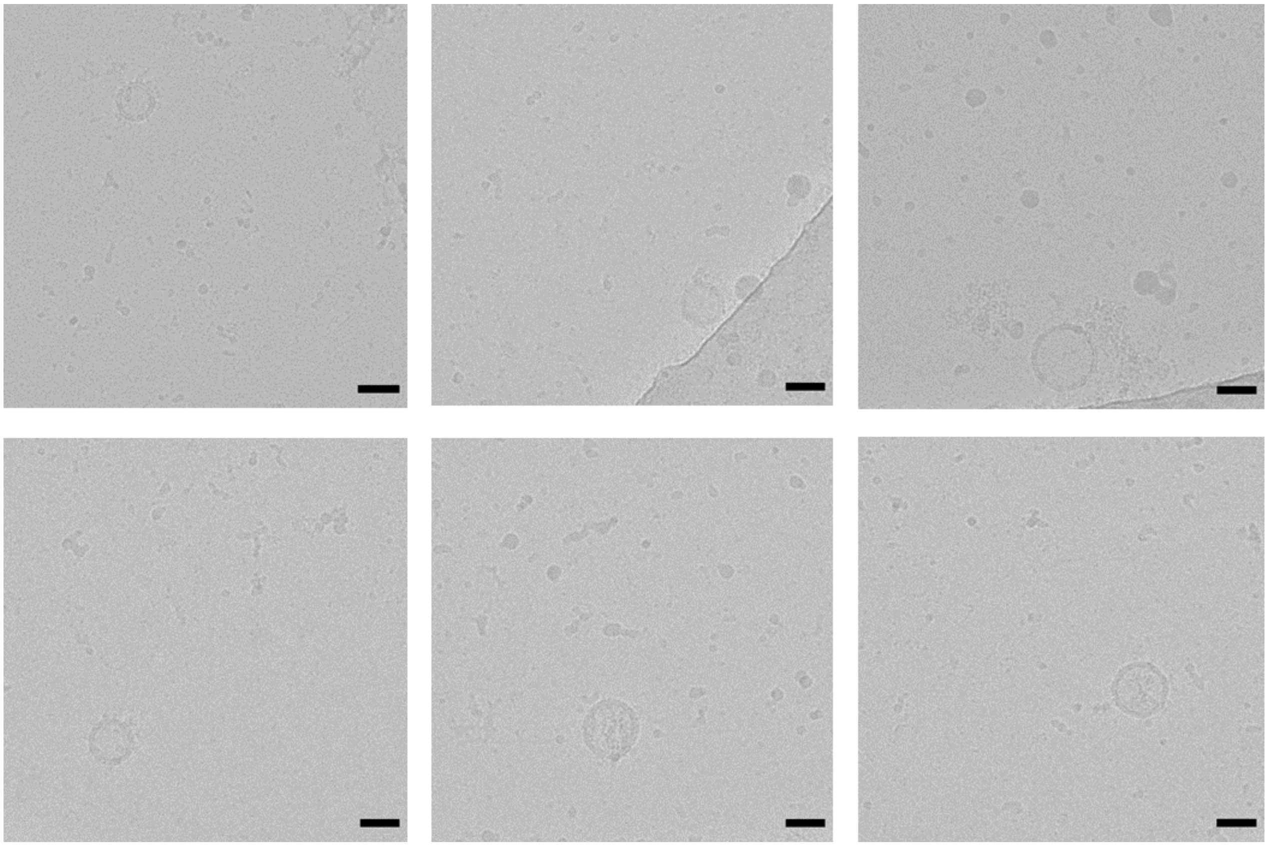

Figure S1. Representative EV imaging of PDEVs by cryo-electron microscopy, scale bar: 100nm.

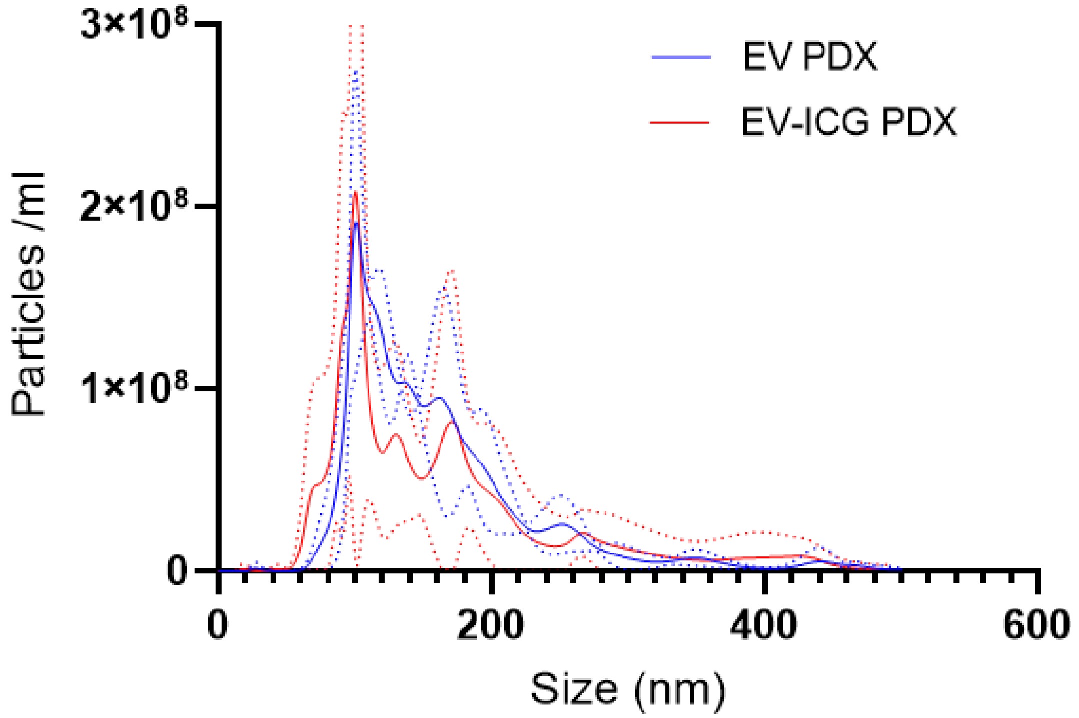

Figure S2. Comparison of NTA profiles of patient-derived EVs before (blue line) and after (red line) ICG loading. The graph reports the mean (solid line) and the S.E.M. (dotted line) of n=6 measures for each experimental group. ICG encapsulation did not significantly change the size distribution profile of EV nanoparticles.

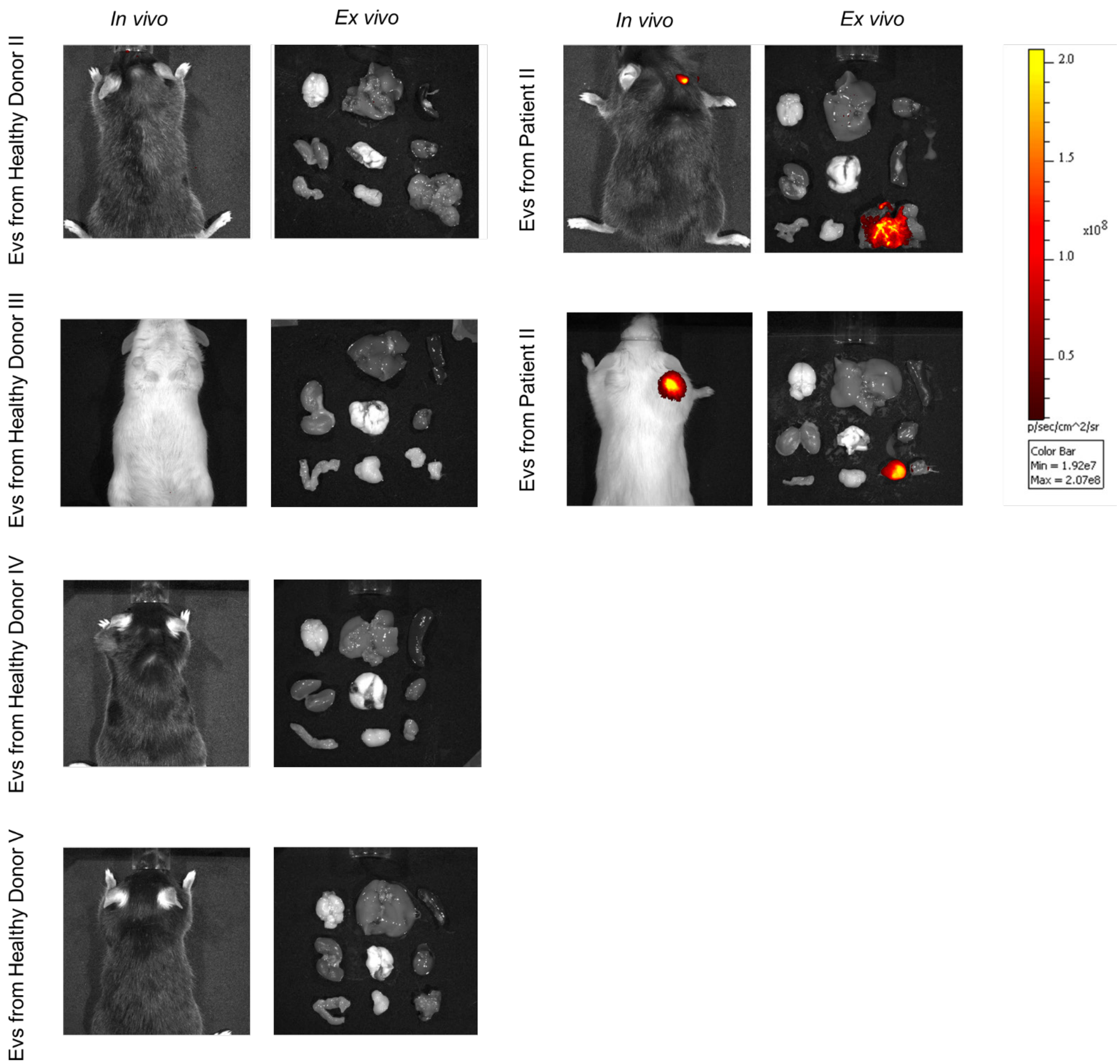

Figure S3. Pseudo-color representative images of ICG fluorescence. *In-vivo* and *ex vivo* imaging of MC-38 tumor-bearing mice (black mice) or mice bearing mammary cancer (NeuT, white mice) injected with EVs obtained from heathy volunteers (left panels) or patient-derived EVs (right panels), each labeled with ICG. Patients-derived EVs showed tropism for MC-38 tumor and for the mammary cancer originated in the NeuT mice: a heterologous (independent of the tumor type) recognition ability of neoplastic tissue as previously reported for cancer-derived EVs (see reference 16 in the main text). No signal could be detected in both mouse model of cancer when injected with ICG-labelled EVs isolated from healthy volunteers.

A

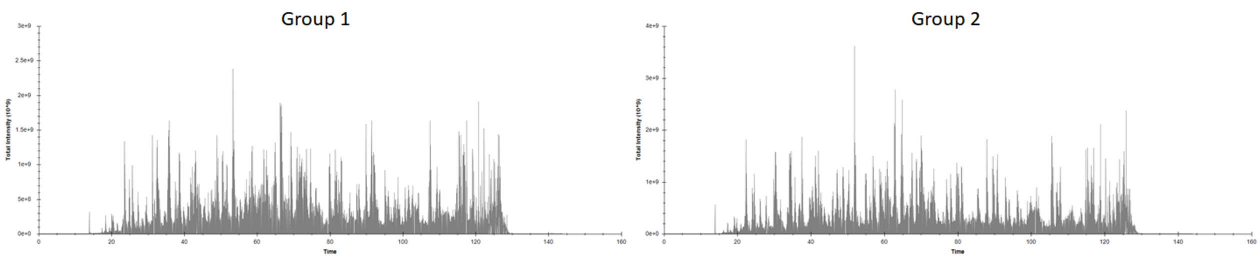

B

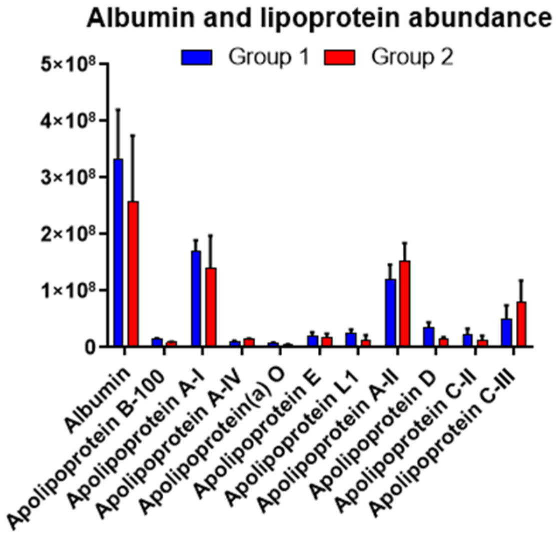

Sidak's multiple comparisons test	Mean Diff,	95,00% CI of diff,	Significant?	Adjusted P Value
Group 1 - Group 2				
Albumin	74000000	-14413179 to 162413179	No	0,1661
Apolipoprotein B-100	5620000	-82793179 to 94033179	No	>0,9999
Apolipoprotein A-I	30066667	-58346512 to 118479845	No	0,9847
Apolipoprotein A-IV	-4073333	-92486512 to 84339845	No	>0,9999
Apolipoprotein(a) O	2513333	-85899845 to 90926512	No	>0,9999
Apolipoprotein E	3466667	-84946512 to 91879845	No	>0,9999
Apolipoprotein L1	11393333	-77019845 to 99806512	No	>0,9999
Apolipoprotein A-II	-32233333	-120646512 to 56179845	No	0,9742
Apolipoprotein D	20966667	-67446512 to 109379845	No	0,9993
Apolipoprotein C-II	11103333	-77309845 to 99516512	No	>0,9999
Apolipoprotein C-III	-29766667	-118179845 to 58646512	No	0,9858

Figure S4. (A) LC chromatographic profiles of protein content in preparations of EVs from CRC patients (left panel) or healthy donors, did not show significant differences in the abundance of the most represented proteins. (B) MS quantification of albumin and lipoprotein content in CRC patients (blue bars) or healthy donors (red bars) did not detect significant differences between the two groups, as assessed by statistical analyses reported in the right panel.

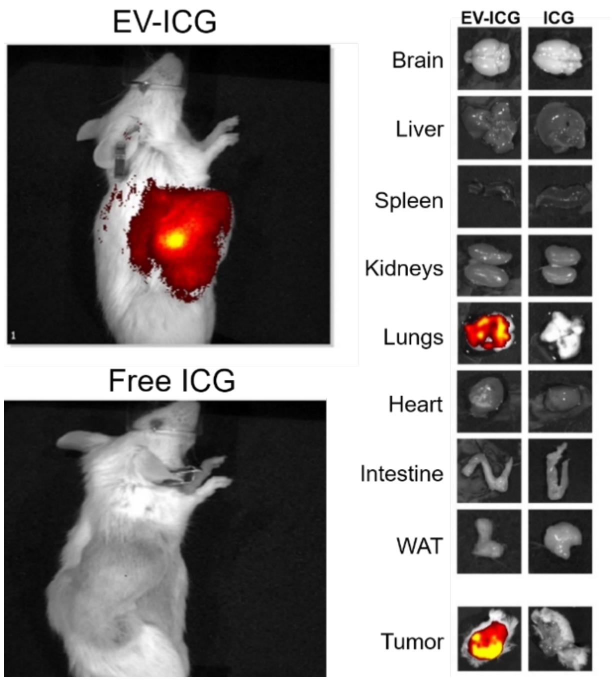

Figure S5. Pseudo-color representation of ICG fluorescence. *In vivo* (left panels) and *ex vivo* (right panels) imaging of PDX mice injected with *i*) patient-derived EVs labeled with ICG (EV-ICG) or *ii*) free ICG ((130 nmol, the same amount included as cargo into the patient-derived EVs).

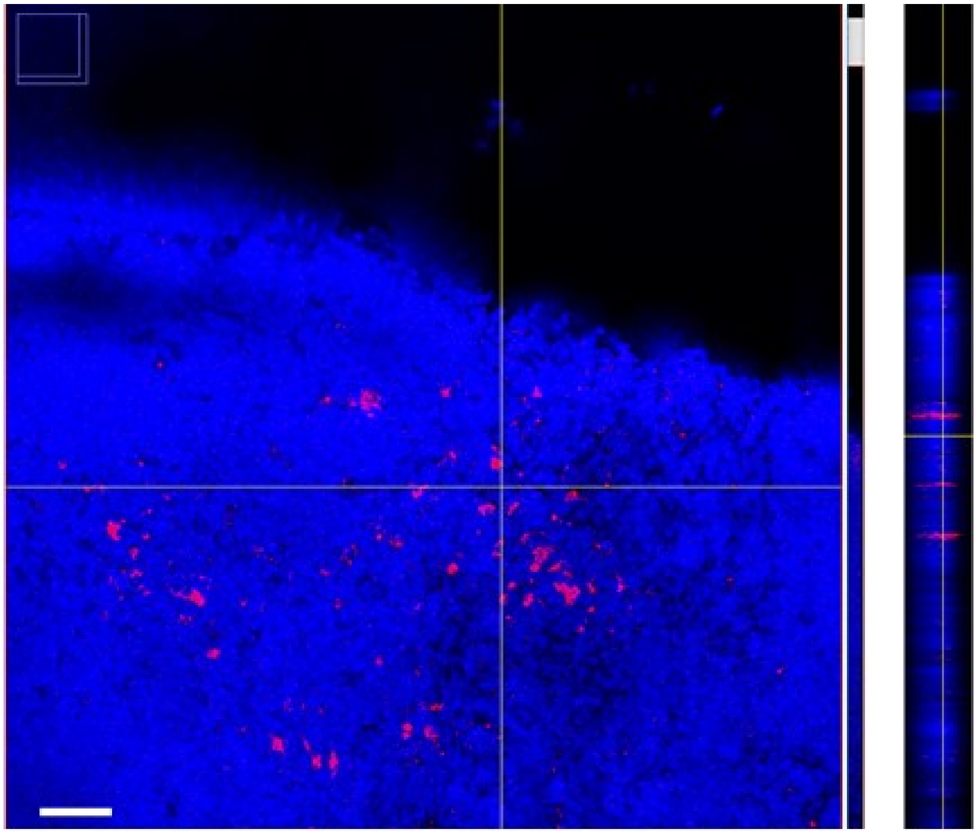

Figure S6. Picture of tumor tissue dissected from PDX II mouse, acquired by confocal laser scanning microscopy (CLSM) and showing the fluorescent signals of nuclei stained with DAPI (blue) and the fluorescence released by ICG (red). In order to assess the distribution of ICG fluorescence in the tissue sections, optical sectioning was performed by acquiring a series of focal planes distant 1 μm along the optical (z) axis of the microscope, using the z-scan mode available on Nikon A1R laser scanning confocal microscope. Orthogonal projections were then reconstructed using ImageJ software (NIH). Briefly, the software projected on the y axis the fluorescent signal acquired in each optical section, simulating a lateral view of the tissue slice to reveal the interior configuration of the imaged region (right panel). (Scale bar: 100 μm)

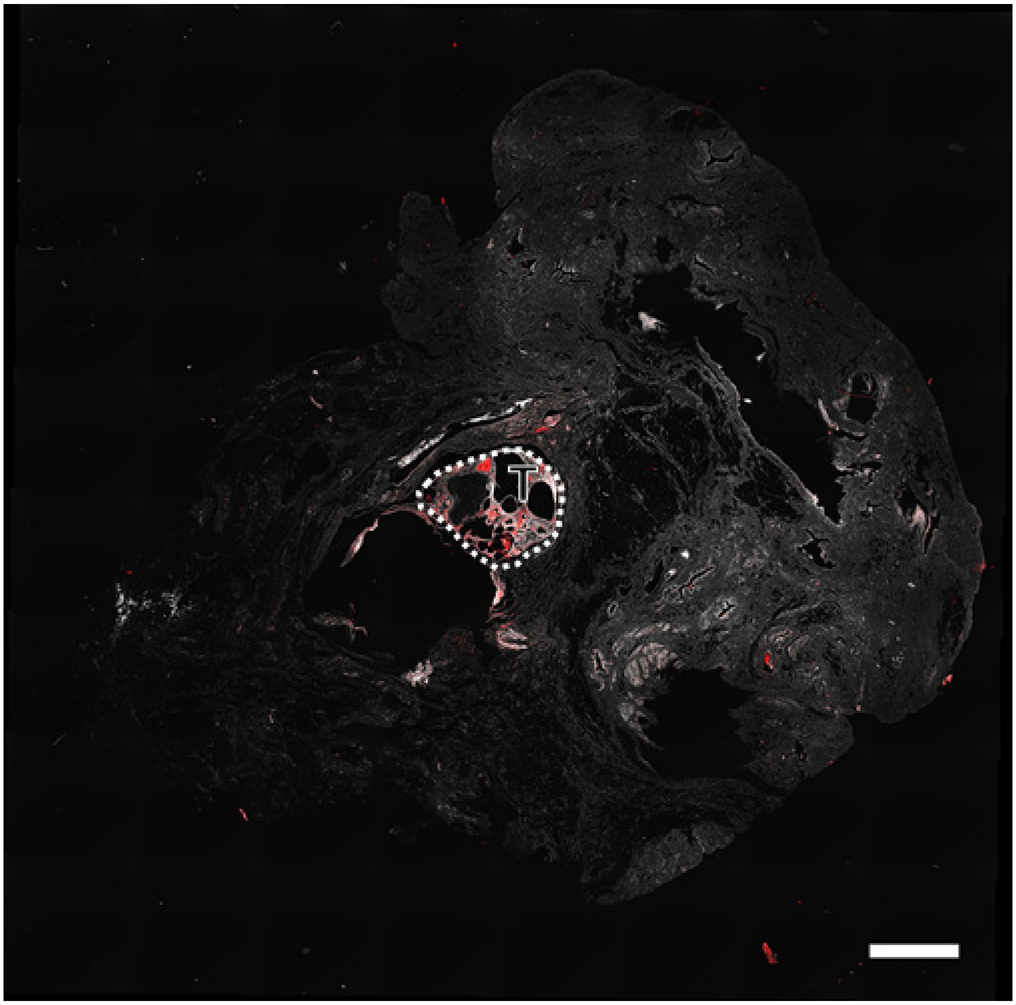

Figure S7. Low-power view of canine mammary-gland tumor specimen classified as carcinoma, represented as a superposition of bright-field (gray) and ICG fluorescence (red). The picture shows the accumulation of fluorescent signal in the tumor tissue (T). Details of the tumor area are reported in Fig. 6B. Scale bar: 2 mm.

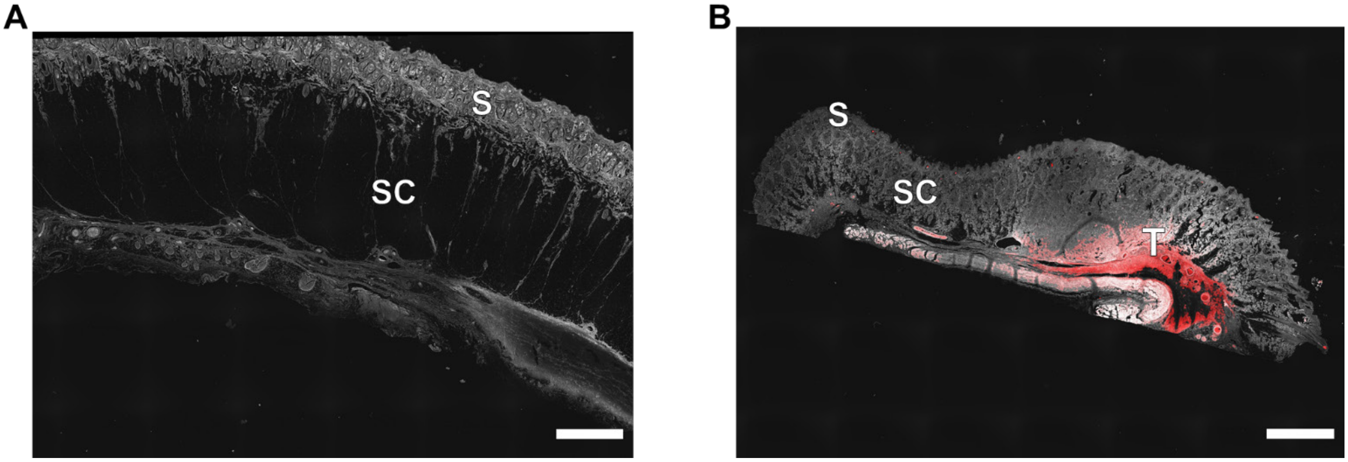

Figure S8. Superposition of bright-field (gray) and ICG fluorescence (red) microscopy images, captured by confocal microscopy from canine specimens obtained from a tumor (mastocytoma) patient. Picture represents the resection margin within a region of normal tissue (A) or region within the mastocytoma tissue (B) of the same patient. S = skin; SC = subcutis; T = tumor (Mast Cell Tumor). Scale bar: 2mm. While samples collected in the non-tumor, cancer-free tissue did not show detectable specific fluorescent emission (A), neoplastic tissue (T) exhibited fluorescence in the NIR spectrum related to ICG (B) thus corroborating the evidence that, also in canine large breeds with spontaneous tumors, a specific labeling of the autologous neoplastic tissue can be obtained with the fluorescent dye conveyed by PDEVs.

Table S1. Patients and tumors characteristics of CRC-bearing patients recruited for PDX generation.

Patient number	Age	Sex	Primary tumor (previously removed)	Stage	Histology Genotype	Serum Markers
**I**	51	F	Left colon (sigmoid)	pT4a, N1a, M1 Synchronous liver M+	Adenocarcinoma G2 RAS/BRAF wild type	CEA: 1.5 CA 19.9: 24
**II**	63	M	Right colon	pT1, N1, M0 Metachronous liver M+	Adenocarcinoma G2 KRAS: mutated (G12V)	CEA: 5.69
**III**	69	M	Right colon	pT3, N1a, M1 Synchronous liver M+	Adenocarcinoma G2 KRAS: mutated (Codon 13)	CEA: 4.40 CA 19.9: 8.4
**IV**	73	F	Right colon	pT4, N0, M0 Metachronous liver M+	Adenocarcinoma G2 KRAS wild type	CEA: 1.5
**V**	54	F	Left colon (sigmoid)	pT2, N0, M0 Metachronous liver M+	Adenocarcinoma G2-G3 KRAS wild type	CEA: 2.05 CA 19.9: 8.0
**VI**	70	F	Right colon	pT3, N0, M1a Synchronous liver M+	Adenocarcinoma G2 RAS/BRAF wild type	CEA:2.84 CA 19.9: 18.4
**VII**	77	F	Right colon	pT3, N1, M1 Synchronous liver M+	Adenocarcinoma G3 BRAF mutated (V600E) KRAS/ NRAS wild type	CEA: 5.2
**VIII**	70	M	Left colon (sigmoid)	pT3, N2, M1 Synchronous liver M+	Adenocarcinoma G2-G3 RAS/BRAF wild type	CEA: 51

Table S2. Results of blood tests on specific panels for testing bone marrow, liver and kidney functions before and after EV injection. No significant variations were observed at early intervals after ICG-loaded EVs.

		**Pre- ICG-loaded EVs injection**		**Post- ICG-loaded EVs injection (24hrs)**	
**Test**	**Unit**	**Dog 1**	**Dog 2**	**Dog 1**	**Dog 2**
**RBC**	n/µL	6.23	5.06	6.69	4.53
**WBC**	n/µL	7.75	16.41	7.70	8.83
**ALP**	U/L	74	231	71	197
**ALT**	U/L	57	70	48	67
**Creatinine**	mg/dL	1.3	0.5	1.0	0.4
**Urea**	mg/dL	64	17	36	24
